# The development of clinical guidelines in China: insights from a national survey

**DOI:** 10.1186/s12961-021-00799-7

**Published:** 2021-12-23

**Authors:** Yang Song, Jing Li, Yaolong Chen, Ruixia Guo, Pablo Alonso-Coello, Yuan Zhang

**Affiliations:** 1 Department of Gynaecology, First Affiliated Hospital of Zhengzhou University, Zhengzhou, China; 2 Iberoamerican Cochrane Centre – Department of Clinical Epidemiology and Public Health, Biomedical Research Institute Sant Pau (IIB Sant Pau), Barcelona, Spain; 3 Vall d’Hebron University Hospital Research Institute (VHIR), Universitat Autònoma de Barcelona, Barcelona, Spain; 4 Evidence-Based Medicine Center, School of Basic Medical Sciences, Lanzhou University, Lanzhou, China; 5 WHO Collaborating Centre for Guideline Implementation and Knowledge Translation, Lanzhou, China; 6 Centro de Investigación Biomédica en Red de Epidemiología y Salud Pública (CIBERESP), Madrid, Spain; 7 Department of Health Research Methods, Evidence, and Impact (HEI), McMaster University, Hamilton, Canada

**Keywords:** Practice guideline, Surveys and questionnaires, Evidence-based practice, China

## Abstract

**Background:**

Previous research suggests that the quality of clinical guidelines (CGs) in China is suboptimal. However, little is known about the methodology that CGs follow. We conducted a national survey of methods used by Chinese CG developers for CG development, adaptation, and updating.

**Methods:**

We used a previously piloted questionnaire based on methodologies of CG development, adaptation, and updating, which was distributed during September–November 2020 to 114 organizations identified from published Chinese CGs (searched 2017–2020), recommended by Chinese CG developers, and recommended by clinical discipline experts.

**Results:**

We collected 48 completed questionnaires (42.1% response). Most organizations developed CGs based on scientific evidence (89.6%), existing CGs (75%), or expert experience and opinion (64.6%). Only a few organizations had a specific CG development division (6.3%), a CG monitoring plan (on clinicians 33.3%; on patients 18.8%), funding (33.3%), or a conflict-of-interest (COI) management policy (23.4%). Thirty (62.5%) organizations reported using a CG development methodology handbook, from international organizations (14/30, 46.7%), methodology or evaluation resources (3/30, 10.0%), expert experience and opinion (3/30, 10.0%), or in-house handbooks (3/30, 10.0%). One organization followed a published adaptation methodology. Thirty-eight organizations (88.4%) reported de novo CG development: 21 (55.3%) formed a CG working group, and 29 (76.3%) evaluated the quality of evidence (21 [72.4%] using a methodological tool). Nineteen organizations (52.8%) reported CG adaptation: three (31.6%) had an adaptation working group, and 12 (63.2%) evaluated the quality of source CGs (2 (16.7%) using the AGREE II instrument). Thirty-three organizations (68.8%) updated their CGs, seven (17.5%) using a formal updating process.

**Conclusions:**

Our study describes how CGs are developed in a middle-income country like China. To ensure better healthcare, there is still an important need for improvement in the development, adaptation, and updating of CG in China.

**Supplementary Information:**

The online version contains supplementary material available at 10.1186/s12961-021-00799-7.

## Background

A clinical guideline (CG) is defined by the Institute of Medicine as “a statement that includes recommendations intended to optimize patient care that is informed by a systematic review of evidence and an assessment of the benefits and harms of alternative care options” [1]. CGs are increasingly used to provide guidance for clinical practice, public health, and policy recommendations [2]. The goal of CGs is to improve clinical practice, minimize unjustified variations in clinical practice, and ensure effective use of healthcare resources [3]. However, developing CGs is a complex and time-consuming process that requires material resources and expert personnel [2, 4]. If resources to develop a high-quality CG are unavailable, adaptation is an alternative [5, 6].

In low- and middle-income countries, the lack of appropriately developed CGs to assist healthcare practice is resulting in suboptimal clinical practice [7, 8]. Although reviews of CGs show that their methodological quality has improved in the past decade [9, 10], in China the quality of CGs continues to be inferior [11–16]. The evidence shows that Chinese CG quality, as assessed by the Appraisal of Guidelines for Research and Evaluation II (AGREE II) instrument [17], is scored at under 30% in most domains [16]. A 2015 study of 109 Chinese CGs reported that only a handful were developed based on research evidence (16; 14.7%), while even fewer critically assessed the certainty of evidence (14; 12.8%) or the strength of recommendations (13; 11.9%) [12].

Empirical evidence shows that China lacks high-quality clinical and epidemiological studies or other types of studies as evidence-based resources [18], which may hinder the adequate updating of Chinese CGs or adaptation for local use. Factors that could influence recommendations or informed decision-making, including resources, cost, feasibility, applicability, and equity, are seldom considered in Chinese CG development processes [11, 14, 15, 19]. Lack of proper incorporation of cost and other considerations potentially hinders adherence to Chinese CGs, but may also contribute to the documented tense relationship between doctors and patients [20]. Furthermore, the development process underlying some Chinese CGs based on existing CGs is also unclear. While previous evidence shows that many international CGs have been used to develop Chinese CGs [12]—for example, the National Comprehensive Cancer Network (NCCN) guidelines or National Institute for Health and Care Excellence (NICE) guidelines—how those source CGs were evaluated and adapted is poorly reported [21].

One important challenge, in terms of improving CG quality in China is the fact that little information is available on the methodology used by developers. To gain more knowledge on this, we conducted a national survey to collect data on Chinese CG development methods and to understand how CGs are developed, adapted, and updated, therefore providing the basis for future improvements in guideline quality in China.

## Methods

### Aim

This was a cross-sectional online national survey to better understand how CGs are developed, adapted, and updated in China.

### Participants

Participants in our survey were key informants and experts affiliated with CG development organizations and expert committees that have developed Chinese CGs in the past 3 years. We adopted a purposive sampling method to recruit participants [22], identified as follows: (1) corresponding contacts of 74 affiliated CG development organizations extracted from 171 Chinese CGs published between January 2017 and February 2020, and retrieved from a literature search in the China National Knowledge Infrastructure database; (2) recommendations from Chinese CG developers; and (3) recommendations from Chinese clinical discipline experts. If initial contacts were not eligible for participation in the survey, they were asked to recommend an eligible person from their organization. The selection procedure is described in Additional file 1: Appendix 1.

### Questionnaire

We developed a self-administered questionnaire based on several methodological and evaluation resources, including AGREE II [17], Grading of Recommendations Assessment, Development and Evaluation (GRADE) [23], GRADE Evidence to Decision (EtD) frameworks [24], Resource Toolkit for Guideline Adaptation (ADAPTE) [6], and the Checklist for the Reporting of Updated Guidelines (CheckUp) [25]. The questionnaire was drafted by one author (YS) and was subsequently reviewed and modified by two other authors (YZ, PAC). The Chinese version of the questionnaire, also available in English (Additional file 1: Appendix 2), was circulated to the contacts in the participating organizations.

The questionnaire consisted of 45 items in five sections: characteristics of the organization (10 questions), de novo CG development (13 questions), CG adaptation (16 questions), CG updating and monitoring (3 questions), and conflict-of-interest (COI) management and funding (3 questions). A free-text box in 33 items collected additional information and comments.

### Survey

We used online software (http://www.wjx.cn) to design the questionnaire and collect responses. The questionnaire adopted a follow-up question format (only participants who answered “yes” needed to answer further questions) [26] and was piloted with four organizations (one national and three international). We refined the survey based on the feedback from pilot testing, which suggested creating follow-up questions and modifying response categories for optimal understanding and response efficiency. We invited participants through email or WeChat message and provided the following information: (1) a description of the study, (2) the purpose of the survey, (3) the main content of the questionnaire, and (4) instructions on completing the questionnaire. We sent two email reminders a month after delivering the invitation and, where possible, reminded potential participants through a WeChat message. On receiving consent from the participants, we sent the survey link by email or WeChat between July and November 2020 and followed up with up to three email or WeChat reminders.

### Analysis

Descriptive statistics were used to analyse the study data. Absolute frequencies and proportions were calculated for all responses. Depending on the methodology used by organizations, we stratified CG development as de novo or adaptation. CGs used for adaptation purposes are referred to as “source CGs”, and recommendations from source CGs are referred to as “source recommendations”. We hypothesized that using a CG development methodology handbook would be associated with the rigour of the guideline development process. The association was determined by Pearson’s chi-square test or Fisher’s exact test (alpha was set at 0.05). Data were analysed using SPSS version 23.0 statistical software (IBM Corp., Armonk, NY, USA). For qualitative data, one author (YS) coded the data and extracted themes related to CG de novo development or adaptation [27], and another author (JL) double-checked the codes and the corresponding quotations. The most relevant topics raised by respondents in free-text areas of the questionnaire were selected on the basis of consensus among the three authors (YS, YZ, JL).

## Results

**Fig. 1:**
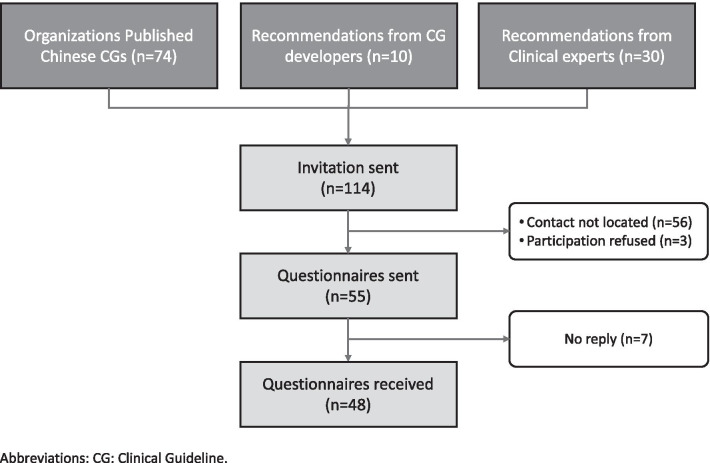
Recruitment flowchart

A total of 114 Chinese CG development organizations and expert committees were contacted by email and WeChat. Responses were received from 55 CG development organizations. After three reminders, we obtained 48 complete responses (42.1% response rate) (Fig. 1) (Additional file 1: Appendix 3).

### Organization characteristics

The organizations, profiled in Table 1, represent six regional economic divisions, 13 provinces, and 13 clinical disciplines as per the Subject Classification of the People's Republic of China [28]. Most respondents worked in hospitals (78.4%), mainly as divisional directors or vice-directors (81.3%). Participating organizations were mostly professional/medical associations (45.8%) or CG expert committees (43.8%). Over half of the organizations (28; 60.6%) had more than 5 years of experience in CG development, and a similar number (30; 62.5%) obtained CG guidance from different resources as their CG development methodology handbook, including international organization or national institute handbooks (46.7%); methodology or evaluation resources such as Guidelines 2.0 checklist, AGREE II, or GRADE (13.3%); in-house handbooks (10.0%); or expert experience and opinion (10.0%). One organization reported following a published adaptation framework—the GRADE EtD frameworks for adoption, adaptation, and de novo development of trustworthy recommendations (GRADE-ADOLOPMENT), specific for CG adaptation. The vast majority of organizations did not have a specific division in charge of CG development (93.8%). Most Chinese organizations developed CGs based on scientific evidence (89.6%), the adaptation of source CGs (75.0%), or expert experience and opinion (64.6%). Organizations that used a CG development methodology handbook were more likely to develop CGs based on scientific evidence (Fisher's exact test; *p* = 0.005) (Additional file 1: Appendix 4).

**Table 1:** Clinical guideline (CG) development organizations and procedures (respondents *n* = 48)

Characteristics	Category	No. (%)
Contact source (*n* = 114)	Published Chinese CG	17/74 (23.0)
	CG developer recommendations	10/10 (100)
	Clinical expert recommendations	21/30 (70.0)
Responder employment^a^ (*n* = 48)	Hospital	40 (78.4)
	Research/knowledge production institution	9 (17.6)
	Government	2 (3.9)
Region^A^ (*n* = 48)	North China	16 (33.3)
	East China	13 (27.1)
	South Central China	12 (25.0)
	Northeast China	3 (6.3)
	Southwest China	2 (4.2)
	Northwest China	1 (2.1)
	Unclear	1 (2.1)
CG scope^B^ (*n* = 48)	Internal medicine	13 (27.1)
	Obstetrics and gynaecology	10 (20.8)
	Clinical epidemiology	5 (10.4)
	Paediatrics	4 (8.3)
	Surgery	4 (8.3)
	Oncology	3 (6.3)
	Acupuncture and tuina science	2 (4.2)
	Geriatrics	1 (2.1)
	Ophthalmology	1 (2.1)
	Nursing	1 (2.1)
	Dermatology and venereology	1 (2.1)
	Pharmaceutics	1 (2.1)
	Chinese medicine	1 (2.1)
	Unknown	1 (2.1)
Organizations	Category	n (%)
Type (*n* = 48)	Professional/medical association	22 (45.8)
	CG expert committee	21 (43.8)
	Research institution	5 (10.4)
Development experience (*n* = 48)	> 10 years	21 (43.8)
	3–5 years	14 (29.2)
	6–10 years	7 (14.6)
	< 3 years	5 (10.4)
	Do not know	1 (2.2)
Use of a handbook^b^ (*n* = 48)	Yes	30 (62.5)
	No	18 (37.5)
Handbook used (*n* = 30)	International organization (e.g., WHO, NICE)	14(46.7)
	Not reported	6 (20.0)
	CG development tool/methodology (e.g., GRADE, AGREE II, or GRADE-ADOLOPMENT)	4 (13.3)
	In-house handbook	3 (10.0)
	Expert experience and opinion	3 (10.0)
Guideline development unit (n = 48)	No	45 (93.8)
	Yes	3 (6.3)
Development process^a^ (*n* = 48)	De novo based on scientific evidence	43 (89.6)
	Adapted from other CGs	36 (75.0)
	De novo based on expert experience and opinion	31 (64.6)
	Adopted directly/translated from other CGs	13 (27.1)
	Updating of other CGs	13 (27.1)

*GRADE* Grading of Recommendations Assessment, Development and Evaluation; *AGREE II* Appraisal of Guidelines for Research and Evaluation II; *NICE* National Institute for Health and Care Excellence

^A^Based on China's regional economic divisions, one participant from abroad collaborates with Chinese CG development

^B^Scope classified according to clinical discipline

^a^More than one response possible

^b^Open-ended response

### CG de novo development

Thirty-eight of 43 organizations (88.4%) reported de novo CG development (Table 2). Only around half of organizations formed a CG working group (55.3%), mainly composed of clinicians (95.2%) and methodologists (85.7%). Most organizations reported conducting a systematic search to retrieve evidence (92.1%), applied eligibility criteria to select evidence (97.4%), assessed the certainty of evidence (94.7%), rated the strength of recommendations (92.1%), and conducted an external review (89.5%). Approximately one out of four organizations that reported having conducted a systematic search did not implement a rigorous search strategy or search in more than two databases, and although most organizations used the GRADE approach to rating the certainty of evidence (92.1%) and the strength of recommendations (89.5%), only around 70% assessed the risk of bias or methodological limitations (a key domain in the GRADE Confidence in the Evidence from Reviews of Qualitative Research (GRADE-CERQual) approach) [29], while 27.6% of organizations evaluated evidence limitations without using any methodological tool.

**Table 2:** De novo clinical guideline (CG) development (n = 38)

Methods (yes responses)	n (%)
The institution has a formal CG working group	21 (55.3)
Evidence is retrieved using systematic searching	35 (92.1)
Eligibility criteria are used to select evidence	37 (97.4)
Evidence limitations are assessed	29 (76.3)
Evidence quality/certainty is rated	36 (94.7)
Strength of recommendations is rated	35 (92.1)
A formal decision-making process is followed	29 (76.3)
The balance between benefits and harms is considered	31 (81.6)
Patient values and preferences are considered	33 (86.8)
Cost and resources needed are considered	33 (86.8)
Other factors are considered	31 (81.6)
An external review is conducted	34 (89.5)

Specific methods (open-ended responses)		n (%)
Stakeholder involvement^a^		
Stakeholders	Working group (*n* = 21)	External review (*n* = 34)
Clinicians	20 (95.2)	34 (100.0)
Methodologists	18 (85.7)	30 (88.2)
Policy-makers	7 (33.3)	18 (52.9)
Patient representatives	9 (42.9)	9 (26.5)
Other	1 (4.8)	4 (11.8)
Systematic search^a^ (*n* = 35)		
Search is conducted in at least two databases		27 (77.1)
Formal/rigorous search strategy is used		26 (74.3)
Other		1 (2.9)
Evidence limitations (*n* = 29)		
Methodological tools (e.g., Cochrane RoB, ROBINS I)		21 (72.4)
Expert opinion		8 (27.6)
Formal decision-making^a^ (*n* = 29)		
Voting system		16 (55.2)
Delphi consensus		15 (51.7)
Informal consensus or expert opinion		8 (27.6)
Cost/resources^a^ (*n* = 33)		
Based on expert opinion		26 (78.8)
Based on evidence synthesis		19 (57.6)
Based on studies (e.g., cost-effectiveness, cost–utility, budgetary impact)		14 (42.4)
Other factors^a^ (*n* = 31)		
Based on expert opinion		26 (83.9)
Based on evidence synthesis (e.g., local data)		16 (51.6)
Based on studies (e.g., interviews)		9 (29.0)
Patient values/preferences^a^ (*n* = 33)		
Based on expert opinion		21 (63.6)
Based on consultation with patient representatives		14 (42.4)
Based on evidence synthesis		12 (36.4)
Based on studies (e.g., reviews, surveys)		9 (27.3)

*RoB* risk of bias; *ROBINS I* Risk of Bias in Non-randomised Studies of Interventions

^a^More than one response possible

**Fig. 2:**
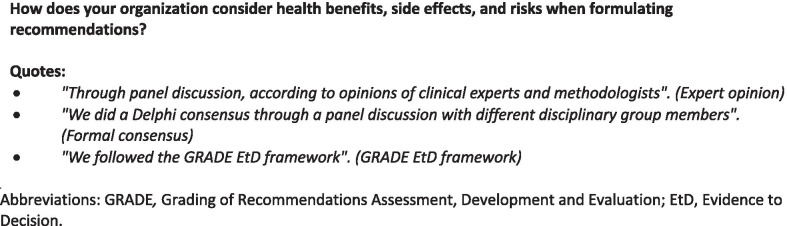
Relevant quotes regarding de novo clinical guideline (CG) development

Twenty-nine (76.3%) organizations formulated recommendations based on a formal decision-making process, whether voting (55.2%), using Delphi consensus (51.7%), or based on expert opinion (27.6%) (Table 2). Organizations that reported using a CG development methodology handbook were more likely to use a formal decision-making process (Fisher's exact test; *p* = 0.009) (Additional file 1: Appendix 4). When formulating recommendations, most organizations reported considering the balance between benefits and harms (81.6%), patient values and preferences (86.8%), cost and resources (86.8%), and other factors (81.6%) (e.g., equity, acceptability, and feasibility). The basis for formulating recommendations varied from expert opinion to the use of research evidence (Table 2, Fig. 2). The reasons for not considering specific aspects were lack of knowledge or expertise.

### CG adaptation

Nineteen of 36 organizations developing CGs through guideline adaptation (52.8%) reported a CG adaptation process (Table 3). Six organizations (31.6%) had an adaptation working group, mainly composed of clinicians (83.3%) and methodologists (83.3%). Most organizations conducted a systematic search to retrieve source CGs (84.2%) and conducted an external review (94.7%). About one in five organizations that conducted a systematic search ultimately did not implement a rigorous search strategy. Eligibility criteria were applied to selecting source CGs by 13 organizations (68.4%), with those who used a CG development methodology handbook being more likely to use a formal eligibility procedure (Fisher's exact test; *p* = 0.007) (Additional file 1: Appendix 4).

**Table 3:** Clinical guideline (CG) adaptation (n = 19)

Methods (yes responses)	n (%)
The institution has a formal CG adaptation working group	6 (31.6)
Evidence is retrieved using systematic searching	16 (84.2)
Eligibility criteria are used to select source CGs	13 (68.4)
Source CG quality is assessed	12 (63.2)
Source CG currency is assessed	19 (100.0)
Source CG recommendations are assessed	14 (73.7)
Source CG recommendation inconsistency is assessed	12 (63.2)
Population differences with source CGs are addressed	16 (84.2)
Health system differences with source CGs are addressed	14 (73.7)
Clinical practice differences with source CGs are addressed	12 (63.2)
Patient values and preferences are considered	18 (94.7)
Cost and resources needed are considered	18 (94.7)
Constraints/barriers are considered	16 (84.2)
Other factors are considered	17 (89.5)
An external review is conducted	18 (94.7)

Specific methods (open-ended responses)		n (%)	
Stakeholder involvement^a^			
Stakeholder	Working group (*n* = 6)	External review (*n* = 18)	
Clinicians	5 (83.3)	18 (100.0)	
Methodologists	5 (83.3)	13 (72.2)	
Policy-makers	3 (50.0)	10 (55.6)	
Patient representatives	1 (16.7)	4 (22.2)	
Other	0 (0.0)	3 (27.8)	
Systematic search^a^ (*n* = 16)			
Search is conducted in at least two databases		14 (87.5)	
Formal/rigorous search strategy is used		12 (75.0)	
Source CG quality^a^ (*n* = 12)			
Expert opinion		8 (66.7)	
Methodological tools (e.g., AGREE II)		2 (16.7)	
Source CG content (*n* = 14)^a^			
Summary tables		11 (78.6)	
Other		3 (21.4)	
Recommendations matrix		0 (0.0)	
Cost/resources^a^ (*n* = 18)			
Based on studies (e.g., cost-effectiveness, cost–utility, budgetary impact)		14 (77.8)	
Based on expert opinion		13 (72.2)	
Based on evidence synthesis		10 (55.6)	
Other factors^a^ (*n* = 17)			
Based on expert opinion		13 (76.5)	
Based on evidence synthesis (e.g., local data)		11 (64.7)	
Based on studies (e.g., interviews)		7 (41.2)	
Patient values/preferences^a^ (*n* = 18)			
Based on expert opinion		15 (83.3)	
Based on studies (e.g., reviews, surveys)		11 (61.1)	
Based on evidence synthesis		10 (55.6)	
Based on consultation with patient representatives		6 (33.3)	

*AGREE II* Appraisal of Guidelines for Research and Evaluation II

^a^More than one response possible

**Fig. 3:**
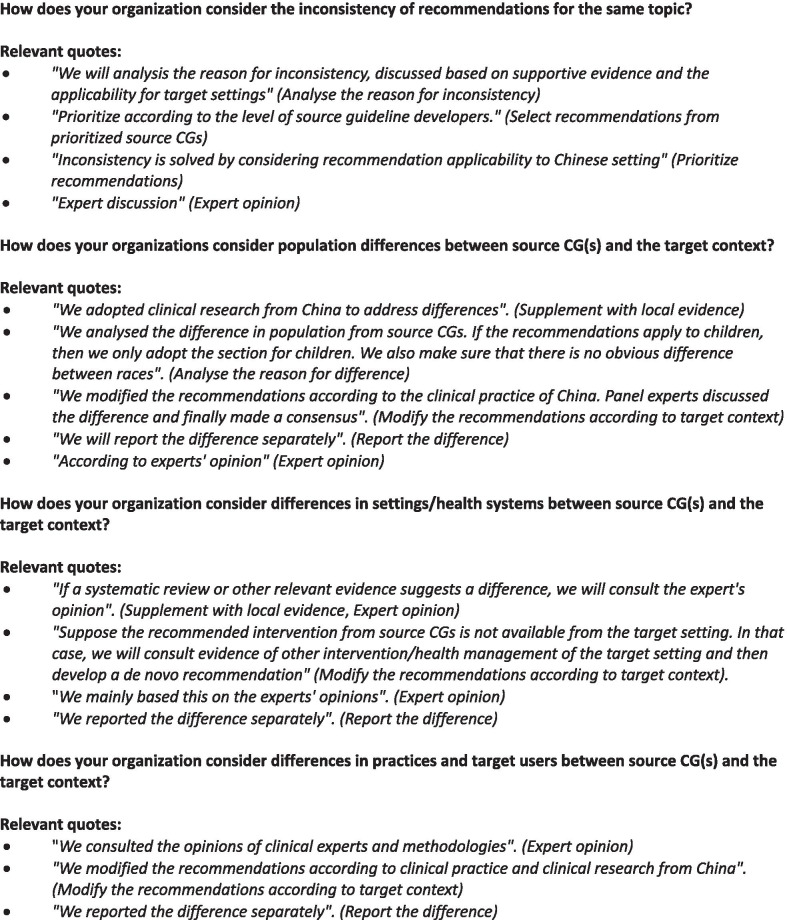
Relevant quotes regarding clinical guideline (CG) adaptation

Over 60% of organizations assessed the source CGs for quality (63.2%), currency (100%), content (73.7%), and inconsistency in source recommendations (63.2%). However, only two organizations (16.7%) used AGREE II to assess the quality of source CGs (the other organizations relied on expert opinion). A summary table was used to assess recommendation content by 11 organizations (78.6%). The methods used to solve source recommendation inconsistency included (1) analysing the reason for an inconsistency, (2) selecting recommendations from prioritized source CG or based on the applicability of the recommendations to the target setting, and (3) discussion among experts (Fig. 3).

In relation to contextualization, most organizations took into consideration differences between the target setting and the source CG setting, including 16 (84.2%) population differences, 14 (73.7%) health system differences, and 12 (63.2%) clinical practice differences. Approaches to contextualizing source CG recommendations included (1) analysing the reason for differences, (2) supplementing with local evidence, (3) considering expert opinion, and (4) modifying recommendations according to the target context (Fig. 3). In the case that differences could not be solved, reporting differences was considered.

Most organizations reported considering patient values and preferences (94.7%), cost and resources (94.7%), constraints or barriers for implementation (84.2%), and other factors (89.5%). As with de novo CG development, the basis for formulating recommendations varied from expert opinion to considering research evidence (Table 3). The reasons for not considering specific aspects were lack of knowledge or expertise.

### CG updating and plans to investigate adherence

Thirty-three of 48 (68.8%) organizations reported having an updating strategy for their CGs, with seven of them (17.5%) confirming a formal updating process (Table 4). Around 60% of the organizations reported an updating frequency of 3–5 years for their CGs. Plans for investigating clinician adherence and target user adherence to CGs were reported by 16 (33.3%) and nine (18.8%) of 48 organizations, respectively.

**Table 4:** Clinical guideline (CG) updating and monitoring (*n* = 48)

Methods (yes responses)	No. (%)
Updating (*n* = 48)	
The institution has a CG updating strategy	33 (68.8)
The institution has a formal CG updating procedure	7 (17.5)
Monitoring (*n* = 48)	
The institution has a plan to check adherence by clinicians	16 (33.3)
The institution has a plan to check adherence by target users	9 (18.8)

Specific methods (open-ended responses)	No. (%)
Updating frequency (*n* = 33)	
3–5 years	22 (66.7)
< 3 years	5 (15.2)
> 5 years	3 (9.1)
Unknown	3 (9.1)

### COI management and funding

Sixteen of 48 (33.3%) organizations reported having received funding for CG development (Table 5). Funding sources included nonprofit associations (50.0%), governments (37.5%), industry (31.3%), medical associations (12.5%), and other sources (18.8%). As for COI management, the type of COI reported included professional or intellectual interests of working group members (27.1%), and financial interests of organizations (6.3%) or of working group members (8.3%). A specific COI policy was reported by 11 (23.4%) organizations.

**Table 5:** Clinical guideline (CG) conflict-of-interest (COI) management and funding (*n* = 48)

Methods (yes responses)	No. (%)
COI management and funding (*n* = 48)	
The institution has funding for CG development	16 (33.3)
The institution has a COI management policy	11 (23.4)
Specific methods (open-ended responses)	n (%)
Funding source (*n* = 16)	
Nonprofit association	8 (50.0)
Government	6 (37.5)
Industry	5 (31.3)
Medical association	2 (12.5)
Other	3 (18.8)
COI types (*n* = 48)	
No COI	33 (68.8)
Professional and intellectual interests of working group members	12 (27.1)
Financial interests of working group members	4 (8.3)
Financial interests of institution	3 (6.3)

## Discussion

### Main findings

Our study describes the current CG development process in China, including de novo development, as well as adaptation and updating practices. While CG development in China is broadly in line with international standards, the methods used for specific steps tend to be both variable and informal. CG development is based on varied sources of CG development methodology handbooks and even expert experience and opinion; many developers perform only informal quality assessment of evidence or of source CGs; few organizations have specific CG development divisions, multiple stakeholder engagement, formal updating systems, a COI policy, or funding to support CG development. Similarly, standard methods are not used to adapt source CGs, even though CGs have been adapted for many years in China.

### Our study in the context of previous research

Our findings, compared to those of previous quality assessment studies, show that the rigour of CG development in China is gradually improving. Zhou et al. (2020), for instance, found that CGs published after 2014 were of significantly higher quality than older CGs [30]. Similarly, a quality assessment of Chinese CGs by Wang et al. [31], published in 2020, reported “rigour of development” scores for CGs published specifically in 2018–2019 that were higher (65.1%) than the overall average median score of below 50%. A new series regarding the development process of evidence-based medicine and clinical guidelines in China published in the *Journal of Clinical Epidemiology* is also in line with our study findings [32].

Unlike quality assessment studies, our survey identified the methodologies that Chinese CG developers follow, which is not limited to what is reported. The reporting of Chinese CGs is very suboptimal as assessed by the Reporting Items of Practice Guidelines in Healthcare (RIGHT) statement [33–35]. Considering that the completeness of reporting impacts quality assessment results for CGs, the assessment scores based on AGREE II are likely to be lower, thereby underestimating the methodological quality of Chinese CGs. Moreover, around 2 years is needed to develop a CG; hence, previous assessment studies reporting poor quality in the AGREE II “rigour of development” domain with the last search date around 2019 or earlier may reflect CG development in or before 2017 [30, 31].

Although the rigour of CG development in China is improving, the methods used vary widely. More than 30% of Chinese CG development organizations in our study did not follow any handbooks or guidance on developing CGs, and the handbooks they used were not only standards from different international organizations, but also methodological tools or expert experience and opinion. Given that evidence rating systems and decision-making procedures vary across international organizations, such discrepancies introduce variability in the Chinese CG development process. NCCN, for instance, uses a different evidence rating system from that used by WHO [36], while NICE also has its own decision-making procedure [37]. In addition, the methods used for specific steps, such as assessing the limitations of evidence or the quality of source CGs, tend to be informal.

However, as was reported by a previous study [11], most Chinese CG development organizations do not have a specific division or group for CG development; this makes our findings regarding inconsistent CG development methodology handbook use and lack of quality assurance monitoring less surprising. The funding sources for CG development point to the involvement of industry funding and, therefore, of COIs. Without proper COI management policies, the evidence-based framework and credibility of CGs is inevitably hampered [38, 39]. Furthermore, few CG organizations have formal updating or adherence monitoring procedures in place. Although around 20% of recommendations become outdated within 3 years, only 15.2% of organizations update their CGs within this period of time [40]. Another area of concern is that most organizations mainly rely on clinicians and so lack participation by other stakeholders, such as patient representatives and policy-makers. Stakeholder engagement is essential for improving CG recommendation uptake and implementation, which should be considered during the CG development process [4, 41]. A lack of stakeholder engagement may lead to controversy and uncertainty, thereby hindering CG implementation [42].

We found that 75% of Chinese CG organizations developed CGs by adapting source CGs, which highlights the widespread use of CG adaptation in China. However, precisely how CG adaptation methods are used is unclear. Of the CG organizations in our study that adapted source CGs, only half reported their adaptation process, and hardly any mentioned following a published adaptation methodology. In addition, as happens with de novo CG development, CG adaptation is informal and lacks monitoring. Only six CG organizations in our study had created an adaptation working group, and only two mentioned having used a validated tool to evaluate the quality of source CGs. Since the quality of adapted CG relies mainly on the source CG, this informality undoubtedly contributes to the low quality of Chinese CGs.

### Limitations and strengths

Our study has some limitations. First, the response rate was relatively low, despite sending two reminders and contacting potential participants using different approaches. However, our sample included 48 Chinese CG development representatives of 13 clinical disciplines and 13 provinces. We did not explore CG development on the basis of consensus, which is yet to be studied and understood.

There are several strengths of our study. First, the survey format with follow-up questions allows us to describe in depth the specific methods used in China and to explore the underlying reasons for the low-level quality of Chinese CGs. Additionally, our study comprehensively describes the CG development process in one middle-income country, including CG de novo development, adaptation, and updating process, which contributes to the improvement of the CG development process as a whole in China. Furthermore, we designed the study questionnaire following international standards and piloted it with both national and international organizations. This allows our methods to provide more reference value to other countries with similar issues.

### Implications for practice and research

CG development in China needs to be standardized. A good CG development process requires a multidisciplinary working group, a rigorous methodology, sufficient and independent funding, sound COI management, and a monitoring and updating system [1]. Stakeholder engagement should be emphasized in the development process of Chinese CGs to ensure that guideline topics are relevant and prioritized and that other factors like acceptability and feasibility are adequately considered, thus facilitating policy-maker adoption of recommendations into policy and practice [43–45]. In addition, sufficient nonprofit public funding and strict COI management strategies should be ensured for CG development, to reduce the potential COI impact on health-related decision-making and clinical practice. Medical associations and government institutions need to assume responsibility for CG monitoring and quality assurance, thereby ensuring the proper implementation of formal development and adaptation methodologies for CGs. CG developers in China need to collaborate closely in standardizing and improving the rigour of CG development, for example, by implementing a standard CG development methodology/handbook and following published reporting guidance such as the RIGHT statement for de novo CGs or CheckUp for updated CGs [25, 46]. Future practices need to build on those aspects so as to improve the quality and reliability of Chinese CGs, and therefore improve healthcare nationwide.

While CG adaptation is an efficient way to develop contextualized recommendations, adapted CGs will only benefit from the quality of source CGs by implementing a rigorous adaptation process [6, 47, 48]. Of the quality published adaptation methodologies available [49], Chinese CG developers could adopt and validate an optimal methodology applicable to the national context. Future research could therefore focus on exploring efficient and rigorous adaptation methods that ensure CG quality and also improve CG implementation.

## Conclusions

CG development and adaptation methodologies, including for updating, as used in China tend to be variable and informal, and so need to be standardized. CG development in general is poorly managed and monitored. Greater effort and more funds need to be invested in improving the quality of Chinese CGs so as to ensure better healthcare.

## Supplementary Information

**Additional file 1.** Appendices.

## Data Availability

All data generated or analysed during this study are included in this published article and its supplementary information files.

## Abbreviations

ADAPTE: Resource Toolkit for Guideline Adaptation
AGREE II: Appraisal of Guidelines for Research and Evaluation II
CheckUp: Checklist for the Reporting of Updated Guidelines
CG(s): Clinical guideline(s)
COI: Conflict of interest
EtD: Evidence to Decision
GRADE: Grading of Recommendations Assessment, Development and Evaluation
GRADE-ADOLOPMENT: GRADE EtD frameworks for adoption, adaptation, and de novo development of trustworthy recommendations
NCCN: National Comprehensive Cancer Network
NICE: National Institute for Health and Care Excellence
RIGHT: Reporting Items of Practice Guidelines in Healthcare
