# Supplementary material for: The development of clinical guidelines in China: insights from a national survey
Source: Health Res Policy Syst. 2021 Dec 23;19:151. doi: 10.1186/s12961-021-00799-7 (PMC8705156; doi:10.1186/s12961-021-00799-7)
Supplement: Supplementary file 1 — Additional file 1. Appendices. [file 12961_2021_799_MOESM1_ESM.docx]

## Appendices

### Appendix 01. Selection of published Chinese clinical guidelines (CGs)

**1. Search strategy**

- **Database:** China National Knowledge Infrastructure
- **From/to:** 2017/01/01 to 2020/02/01
- **Filters:** Health-related; Published in journals
- **Excluded:** National and international conferences, newspapers, master and PhD theses

| #1 | “zhinan” ti |
| --- | --- |
| #2 | “gongshi” ti |
| #3 | “linchuangzhinan” ti |
| #4 | “tuijianyijian”ti |
| #5 | “shijianzhinan” ti |
| #6 | #1 OR #2 OR #3 OR #4 OR #5 |
| #7 | “fenxi” ti |
| #8 | “jiedu” ti |
| #9 | #7 OR #8 |
| #10 | #6 NOT #9 |

**2. Eligibility**

We included CGs that (1) were published in Chinese; (2) had more than one recommendation; (3) reported the name of the development organisation; (4) were developed by Chinese organisations, and (5) were published in the previous 3 years. We excluded CGs that (1) described/analysed CG development methods by using recommendations as samples; (2) were not health-related; (3) were CG quality assessment studies, and (4) were conducted by international organisations (not Chinese organisations).

Search results were screened by two authors (YS, JL) to identify potentially eligible CGs. The same two authors independently confirmed eligibility based on the full-text assessment of potentially relevant CGs. Disagreement was solved by discussion. The main CG information recorded in tabular format included title, publication year, scope, development organisation, contact information, and funding information.

**3. Search results**

The pragmatic search retrieved 3853 hits. After screening titles and abstracts and excluding duplications, 264 full-texts were reviewed. Of these, 171 published Chinese CGs were included in the study, from which 74 CG development organisations and 113 contacts were identified. The Chinese CGs were mainly developed by medical associations (21/74), expert committees (24/74), and working groups (19/74), and, to a lesser degree, by the government (3/74), hospitals (5/74), a journal (1/74), and a research organisation (1/74).

**Figure S1. Study flow chart for the selection of published Chinese clinical guidelines**

### Appendix 02. Questionnaire (translated from Chinese)

| **Section A. Institution characteristics (10 questions)** | | |
| --- | --- | --- |
| A1 | What is the name of your institution? (Please provide the full name) | ( ) |
| A2 | Which kind of organisation is your institution? | a. Public institution (e.g., government); b Hospital; c. Scientific society (e.g., Association of Cardiologists); d. Research institution; e. Private organisation  f. Other (Please specify): （ ） |
| A2.1 | What position do you hold in this institution? | a. Director/Supervisor; b. Deputy Director/Deputy Supervisor; c. Coordinator; d. Working Group Leader; e. Methodology Expert/Consultant; f. Other: ( ) |
| A3 | How many years has your institution been developing guidelines? | a. Less than 3 years; b. 3-5 years; c. 6-10 years；d. More than 10 years; e. Do not know |
| A4 | How many guidelines has your institution developed? | a. None; b. Less than 3; c. 3-5; d. 6-10; e. More than 10; f. Do not know |
| A5 | What is the average size of your organisation's guideline development working group? | a. 1-10 members; b. 11-20 members; c. 20-30 members; d. More than 30 members; e. Do not know |
| A6 | How many guidelines from your institution are collaborations with multiple institutions? | a. ≥50 % developed by collaboration with multiple institutions  b. < 50 % developed by collaboration with multiple institutions |
| A7 | On average, how long does your institution take to develop a guideline? | a. 3-6 months; b. 6-12 months; c. 1 -2 years; d. 2-3 years; e. More than 3 years. |
| A8 | Does your institution reference a handbook/manual to develop clinical practice guidelines? | a. Yes; b. No.  If YES, please specify if published somewhere and, if available online, provide the link:  ( ) |
| A9 | Does your institution have a specific department for guideline development? | a. Yes; b. No.  If YES, please specify the department's name:  ( ) |
| A10 | How does your institution develop a guideline?  (Multiple options: more than one response can be selected) | a. De novo guidelines based on a systematic review of the scientific evidence (e.g., clinical trials, observational studies, etc.)  b. De novo guidelines are not based on scientific evidence but just expert opinion.  c. Adapted guidelines from other previously published guidelines (e.g., a NICE guideline)  d. Adopted or translated guidelines from other guidelines  e. Updating of other guidelines.  If you use other methods, please specify: ( ) |
| A 10.1 | Please respond according to your guideline development process (A10): | a. Development of de novo guidelines, based on evidence or consensus (please answer Part B)  b. Adaptation/adoption/translation of existing/source guidelines, or development of de novo guidelines based on existing/source guidelines (please answer Part C)  c. Both de novo development and adaptation/adoption/translation (please answer both parts B and C) |
| **If your institution develops de novo guidelines, please go to section B.**  **If your institution adapts/adopts/translates other sources/original guidelines, please go to section C.**  **If your institution both develops de novo and adapts/adopts/translates guidelines, please reply to both B and C sections.** | | |
| **Section B. De novo guidelines (13 questions)** | | |
| B1 | Does your institution have a formal panel for each guideline?  (Multiple options: if YES, more than one response can be selected) | a. Yes; b. No.  If YES, which roles below does the guideline development group typically include?  1. Clinicians  2. Patients  3. Methodologists  4. Policymakers  5. Other roles: (please clarify ) |
| B2 | How does your institution choose the scope/key questions?  (Multiple options: more than one response can be selected) | a. The prevalence of the condition  b. The existence of underuse, overuse, or misuse of intervention s  c. The burden associated with the condition (system, financial, or patient burden)  d. Concerns about practice variations  e. Cost associated with different practice options  f. The likelihood that the guideline will be effective in influencing practice  g. The potential for improving quality of care and/or patient outcomes (survival or quality of life) |
| B3 | Does your institution conduct a systematic search to retrieve evidence? | a. Yes; b. No.  If YES, please specify:  1. Searched at least two databases  2. Designed rigorous search strategy  3. Other (please specify) |
| B4 | Do you have criteria for selecting the evidence? | a. Yes; b. No. |
| B5 | Do you evaluate the risk of bias/methodological limitations for the included evidence? | a. Yes; b. No; c. not applicable.  If YES, please specify which methods you used:  1. Using an instrument (e.g., Cochrane handbook risk of bias assessment tool for RCTs, ROBINS2 for non-randomised studies etc.)  2. Consensus assessment  3. Other (please specify or provide the link if available online): |
| B6 | Do you assess the quality of the evidence? | a. Yes; b. No; c. Not applicable.  If YES, please specify which rating system you used  1. Specific rating system (e.g., GRADE)  2. Oxford rating system  3. Other (please specify or provide the link if available online): |
| B7 | Do you grade the strength of recommendations? | a. Yes; b. No; c. Not applicable.  If YES, please specify which rating system you used  1. Specific rating system (e.g., GRADE)  2. Oxford rating system  3. Other (please specify or provide the link if available online: |
| B8 | Do you use a specific method to formulate recommendations? (Multiple options: if YES, more than one response can be selected) | a. Yes; b. No.  If YES, please clarify how you reached the final decisions:  a. Voting system  b. Informal consensus  c. Delphi consensus techniques  d. Glaser techniques  e. Other (please specify or provide the link if available online): |
| B9 | Do you consider health benefits, side effects, and risks when formulating recommendations? | a. Yes; b. No.  If YES, please specify or provide the link if available online (e.g., reference published methodology, through expert consensus, or considering clinical expert and methodologists opinions)  ( )  If NO, please specify the reason:  ( ) |
| B10 | Do you consider patient values and preferences when formulating recommendations? | a. Yes; b. No.  If YES, please clarify how you considered this aspect  1. Using evidence like systematic reviews of patient values and preferences.  2. Conducting systematic reviews or primary studies on patient values and preferences  3. Panel opinion  4. Consultation with patients  5. Other methods (please specify or provide the link if available online): ( )  If NO, please choose a reason below  a. Don't know why  b. Don't know how  c. Less effort plan to put  d. Other reason, please clarify: ( ) |
| B11 | Do you consider cost and resources needed when formulating recommendations? | a. Yes; b. No.  If YES, please clarify how you considered this aspect  1. Through systematic reviews of economic evaluations  2. Conducting de novo cost-effectiveness or cost-utility analysis.  3. Conducting budgetary impact analysis  4. Panel opinion  5. Other methods (please specify or provide the link if available online): ( )  If NO, please choose a reason below  a. Don't know why  b. Don't know how  c. No plan  d. Other reason, please clarify: ( ) |
| B12 | Do you consider other factors when formulating recommendations (e.g., equity, acceptability, and feasibility)? | a. Yes; b. No.  If YES, please clarify how you considered this aspect  1. Using evidence such as systematic reviews or local data.  2. Conducting systematic review or other types of research  3. Panel opinion  4. Other methods (please specify or provide the link if available online):  If NO, please choose a reason below  a. Don't know why  b. Don't know how  c. No plan  d. Other reason, please clarify: ( ) |
| B13 | Are guidelines externally reviewed prior to publication? | a. Yes; b. No.  If YES, which relevant stakeholders are included?  a. Clinical experts  b. Methodological experts  c. Target population (patients, public) representatives  d. Policymakers  e. Other (e.g., journal editors) (please specify or provide the link if available online): ( ) |
| **Section C. Adapted/adopted/translated guidelines (16 questions)** (Note: source CGs means the CGs used for adaption/adoption/translation) | | |
| C1 | Do you have a formal team/group specific for each guideline adaptation? | a. Yes; b. No.  If YES, which roles below do your guideline development group include?  1. Clinicians  2. Patients  3. Methodologists  4. Policymakers  5. Other roles: (please clarify): ( ) |
| C2 | How do you choose the guideline scope/key questions (e.g., population, intervention, control, and outcomes, PICO) before adapting the guideline?  (Multiple options: more than one option can be selected) | a. The prevalence of the condition  b. The existence of underuse, overuse, or misuse of the intervention  c. The burden associated with the condition (such as a system, financial, or patient burden)  d. Concerns about practice variations and whether baseline data on current practice are available.  e. Cost associated with different practice options  f. The likelihood that the guideline will be effective in influencing practice  g. The potential for improving quality of care and/or patient outcomes (such as survival or quality of life)  h. The existence of relevant good-quality evidence-based guidelines |
| C3 | Do you use a systematic search method for source guidelines? | a. Yes; b. No; c. Not applicable (adaptation of specific source CGs)  If YES, please specify:  1. Searched at least two databases  2. Designed a rigorous search strategy  3. Other (please specify): ( ) |
| C4 | Do you have criteria to include/select source guidelines? | a. Yes; b. No; c. Not applicable (adaptation of specific source CGs) |
| C5 | Do you assess the quality of the included source guidelines? | a. Yes; b. No.  If YES, please specify which methods you use to assess the quality of source guidelines  (e.g., evaluation tool, expert opinions, panel discussion, etc.) ( ) |
| C6 | Do you assess the currency of the included source guidelines? | a. Yes; b. No; |
| C7 | Do you assess the content of the included source guidelines using matrices?  (Only when more than one source guideline is included) | a. Yes; b. No.  If YES, which methods do you use?  1. A table format  2. A recommandations matrix  3. Other (please specify): ( ) |
| C8 | Do you assess the consistency of the included source guidelines? | a. Yes; b. No; c. Only one source guideline included, so consistency assessment is not applicable.  If YES, please specify how you address the inconsistency of source recommendations? (please reply according to your institution's method, e.g., how does your institution prioritise specific recommendations or the methods to analyse the inconsistency): ( ) |
| C9 | Do you consider population differences between source guidelines and the target context? | a. Yes; b. No.  If YES, please specify how do you resolve the difference (please reply according to your institution's method, e.g., how does your institution modify the recommendations, etc.):  ( ) |
| C10 | Do you consider setting/health system differences between source guidelines and the target context? | a. Yes; b. No.  If YES, please specify how you resolve the difference (please reply according to your institution's method, e.g., does your institution consider expert opinions or other sources of evidence?):  ( ) |
| C11 | Do you consider practice variations and target user differences between source guidelines and the target context? | a. Yes; b. No.  If YES, please specify how you resolve the difference (please reply according to your institution's method, e.g., does your institution consider expert opinion or other sources of evidence?):  ( ) |
| C12 | Do you consider patient values and preferences of the target context? | a. Yes; b. No.  If YES, please clarify how you considered this aspect  1. Using evidence like systematic reviews of patient values and preferences.  2. Conducting systematic reviews or primary studies of patient values and preferences  3. Panel opinion  4. Consultation with patients  5. Other methods (please specify or provide the link if available online):  ( )  If no, please choose a reason below  a. Don't know why  b. Don't know how  c. No plan  d. Other reason, please clarify: ( ) |
| C13 | Do you consider whether cost and resources are applicable when adapting source guidelines? | a. Yes; b. No.  If YES, please clarify how you considered this aspect  1. Through systematic reviews of economic evaluations  2. Conducting de novo cost-effectiveness or cost-utility analysis.  3. Conducting budgetary impact analysis  4. Panel opinion  5. Other methods (please specify or provide the link if available online): ( )  If no, please choose a reason below  a. Don't know why  b. Don't know how  c. No plan  d. Other reason, please clarify: ( ) |
| C14 | Do you consider other factors when formulating recommendations (e.g., equity, acceptability, and feasibility)? | a. Yes; b. No.  If YES, please clarify how you considered those aspects  1. Using evidence such as systematic reviews or local data.  2. Conducting systematic reviews or other types of research  3. Panel opinion  4. Other methods (please specify or provide the link if available online):  If NO, please choose a reason below  a. Don't know why  b. Don't know how  c. No plan  d. Other reason, please clarify: ( ) |
| C15 | Do you consider how target context constraints/barriers (like legislation, policies, or health care setting resources) might impact implementation? | a. Yes; b. No.  If YES, please clarify how you considered those aspects.  1. Using evidence like systematic reviews or local data.  2. Conducting systematic reviews or other types of research  3. Panel opinion  4. Other methods (please specify or provide the link if available online)::  If NO, please choose a reason below  a. Don't know why  b. Don't know how  c. No plan  d. Other reason, please clarify: ( ) |
| C16 | Are guidelines externally reviewed prior to publication? | a. Yes; b. No.  If YES, which relevant stakeholders are included?  a. Clinical experts  b. Methodological experts  c. Target population (patients, public) representatives  d. Policymakers  e. Other (e.g., journal editors) (please specify or provide the link if available online): ( ) |
| **Section D. Updating and monitoring (3 questions)** | | |
| D1 | Does your institution have an updating strategy for your guideline? | a. Yes; b. No; c. Not sure  D1.1 If YES, what is the update frequency for guidelines?  a. < 3 years; b. 3-5 years.  c. > 5 years; d. Not sure.  D1.2 If YES, is there a formal process to identify whether the guideline is up to date?  a. Yes; b. No; c. Not sure.  If YES, please clarify if it is published somewhere and provide the link if available online:  ( ) |
| D2 | Do you have a plan to investigate the adherence of clinicians to your guidelines? | a. Yes; b. No.  If YES, please clarify or provide the link if available online ( ) |
| D3 | Do you have a plan to investigate the adherence of target users (patients, public) to your guidelines? | a. Yes; b. No.  If YES, please clarify or provide the link if available online ( ) |
| **Section E. Conflict of interest management and funding (3 questions)** | | |
| E1 | Does your institution receive any funding for guideline development? | 1. Yes; 2. No.  If YES, which types of funding does your institution receive?  1. Commercial funding  2. Government funding  3. Non-profit association funding  4. Pharmaceutical funding  5. Other funding, please clarify: （ ） |
| E2 | Does your institution have a policy for managing conflicts of interest? | a. Yes; b. No.  If YES, please clarify or provide the link if available online ( ) |
| E3 | Which kind of conflicts of interest does your guideline normally refer to? | a. Personal financial interests  b. Institutional interests  c. Professional and intellectual interests  d. Clinical income interests  e. Other (please clarify): ( ­­­­­­­­­­­­ ­­­­­­­­­­­­) )  f. No conflict of interest |

### Appendix 03. Participating organisations

| **No.** | **Organisation name** |
| --- | --- |
| 1 | Beijing Children's Hospital affiliated to Capital Medical University, Epidemiology and Evidence-Based Medicine Centre |
| 2 | Beijing Friendship Hospital, Capital Medical University |
| 3 | Beijing University of Chinese Medicine |
| 4 | China Association of Integrated Traditional Chinese and Western Medicine, Respiratory Disease Professional Committee |
| 5 | China Medical Equipment Association, Pharmacy Equipment and Technology Professional Committee |
| 6 | China Welfare Association |
| 7 | Chinese Acupuncture and Moxibustion Society Acupuncture Clinical Branch /Chinese Preventive Medicine Association Evidence-based Medicine Committee, Methodology Group |
| 8 | Chinese Anti-Cancer Association, Gynaecological Oncology Committee Member |
| 9 | Chinese Association of Rehabilitation Medicine, Chinese Maternal and Child Health Association |
| 10 | Chinese Eugenics Science Association/China Healthcare International Exchange Promotion Association |
| 11 | Chinese Medical Association Digestive Endoscopy Branch, Enteroscopy and Capsule Endoscopy Group |
| 12 | Chinese Medical Association Gynaecological Oncology Branch |
| 13 | Chinese Medical Association Henan Provincial Family Planning Committee Specialist Branch |
| 14 | Chinese Medical Association Hepatology Branch |
| 15 | Chinese Medical Association Hepatology Branch, Hepatitis Group |
| 16 | Chinese Medical Association Hepatology Branch, Liver Disease and Artificial Liver Group |
| 17 | Chinese Medical Association Infectious Diseases Branch |
| 18 | Chinese Medical Association Obstetrics and Gynaecology Branch, Menopause Group |
| 19 | Chinese Medical Association Organ Transplantation Branch |
| 20 | Chinese Medical Association Paediatric Branch, Respiratory Specialty Group |
| 21 | Chinese Medical Association Paediatric Branch, Haematology Group |
| 22 | Chinese Medical Association Paediatric Development Committee |
| 23 | Chinese Medical Association Paediatrics Branch, Neonatology Group/Chinese Medical Doctor Association Neonatologists Branch |
| 24 | Chinese Medical Association Perinatal Medicine Branch |
| 25 | Chinese Medical Association Perinatal Medicine Branch, Foetal Medicine Group |
| 26 | Chinese Medical Association Reproductive Branch |
| 27 | Chinese Medical Association Shaanxi Society of Infectious and Parasitic Diseases |
| 28 | Chinese Medical Association Surgery Branch, Breast Surgery Group |
| 29 | Chinese Medical Doctor Association Infectious Physician Branch |
| 30 | Chinese Medical Doctor Association Obstetrics and Gynaecology Branch |
| 31 | Chinese Medical Doctor Association Surgeons Branch, Thyroid Surgeons Committee |
| 32 | Chinese Preventive Medicine Association Women's Health Care Branch, Mammalogy Group |
| 33 | Chinese Research Hospital Association Liver Disease Branch |
| 34 | Chinese Society of Gerontology |
| 35 | Chinese Society of Traditional Chinese Medicine |
| 36 | Chinese Society of Traditional Chinese Medicine Spleen and Stomach Disease Branch |
| 37 | Guangdong Provincial Hospital of Traditional Chinese Medicine |
| 38 | Huashan Hospital affiliated to Fudan University |
| 39 | McMaster University Department of Health Research Methods, Evidence, and Impact (collaboration with the Chinese retina Society) |
| 40 | Nanjing Jinling Hospital |
| 41 | National Cancer Diagnosis and Treatment Quality Control Centre, Radiotherapy Quality Control Expert Committee |
| 42 | Qilu Hospital at Shandong University |
| 43 | Shanghai Changzheng Hospital |
| 44 | Shengjing Hospital affiliated to China Medical University |
| 45 | Society of Integrated Traditional Chinese and Western Medicine Professional Obstetrics and Gynaecology Committee |
| 46 | State Key Laboratory of Ophthalmology |
| 47 | University of Nottingham Ningbo |
| 48 | Zhongnan Hospital at Wuhan University |

### Appendix 04. Clinical guideline (CG) development by reference handbook use

|  | **Use of a handbook** | | **Total** | |
| --- | --- | --- | --- | --- |
|  | **YES** | **NO** |  |  |
|  | n | n | n | *P* |
| CG was developed based on scientific evidence (n=48) |  |  |  |  |
| Yes | 30 | 5 | 35 | 0.005* |
| No | 0 | 13 | 13 |  |
| Funding received for CG development (n=48) |  |  |  |  |
| Yes | 14 | 2 | 16 | 0.011* |
| No | 16 | 16 | 32 |  |
| COI management policy in place (n=48) |  |  |  |  |
| Yes | 11 | 1 | 12 | 0.018* |
| No | 19 | 17 | 36 |  |
| Formal updating procedure in place (n=48) |  |  |  |  |
| Yes | 6 | 1 | 7 | 0.711* |
| No | 11 | 9 | 20 |  |
| Do not know | 13 | 8 | 21 |  |
| Plan in place to investigate CG adherence by clinicians (n=48) |  |  |  |  |
| Yes | 9 | 7 | 16 | 0.527 |
| No | 21 | 11 | 32 |  |
| Plan in place to investigate CG adherence by target users (n=48) |  |  |  |  |
| Yes | 5 | 4 | 9 | 0.711* |
| No | 25 | 14 | 39 |  |
| A formal department exists for CG development (n=48) |  |  |  |  |
| Yes | 3 | 0 | 3 | 0.282* |
| No | 27 | 18 | 45 |  |
| A formal method in place to formulate recommendations (*de novo* CGs) (n=38) |  |  |  |  |
| Yes | 24 | 5 | 29 | 0.009* |
| No | 3 | 6 | 9 |  |
| Eligibility criteria applied to include source CGs (adapted CGs) (n=18)^a^ |  |  |  |  |
| Yes | 10 | 3 | 13 | 0.007* |
| No | 0 | 5 | 5 |  |

Abbreviations: COI, conflict of interest. a One organisation adapted one chosen guideline, and eligibility criteria are not applicable. * Fisher's exact test were used when n < 5.
